# Personalized recommendation model of electronic commerce in new media era based on semantic emotion analysis

**DOI:** 10.3389/fpsyg.2022.952622

**Published:** 2022-07-22

**Authors:** Yuzhi Liu, Zhong Ding

**Affiliations:** School of Joumalism and Communication, Sichuan International Studies University, Chongqing, China

**Keywords:** classified recommendation, deep learning, E-commerce, emotion analysis classified recommendation, emotion analysis

## Abstract

Electronic commerce (E-commerce) through digital platforms relies on diverse user features to provide a better user experience. In particular, the user experience and connection between digital platforms are exploited through semantic emotions. This provides a personalized recommendation for different user categories across the E-commerce platforms. This manuscript introduces a Syntactic Data Inquiring Scheme (SDIS) to strengthen the semantic analysis. This scheme first identifies the emotional data based on user comments and repetition on the E-commerce platform. The identifiable and non-identifiable emotion data is classified using positive and repeated comments using the deep learning paradigm. This classification attunes the recommendation system for providing best-affordable user services through product selection, ease of access, promotions, etc. The proposed scheme strengthens the user relationship with the E-commerce platforms by improving the prioritization of user requirements. The user’s interest and recommendation factors are classified and trained for further promotions/recommendations in the learning process. The recommendation data classified from the learning process is used to train and improve the user-platform relationship. The proposed scheme’s performance is analyzed through appropriate experimental considerations. From the experimental analysis, as the session frequency increases, the proposed SDIS maximizes recommendation by 15.1%, the data analysis ratio by 9.41%, and reduces the modification rate by 17%.

## Introduction

E-commerce platforms are places where an individual can buy and sell products or goods using an internet connection. E-commerce applications make use of software applications that are mainly designed to provide various services for supporting E-commerce among individuals and organizations (Nguyen and Cho, 2020). E-commerce is an emerging technology that increases the efficiency rate in communication services for users. The recommendation model is most commonly used in E-commerce to improve the quality of service (QoS) and the effectiveness of goods (Li et al., 2019). Optimization algorithms are used in the recommendation model, which finds out the details and knowledge of goods to provide an effective dataset for consumers in an online application (Liao et al., 2021). The recommendation model creates a taxonomy to identify the product’s location, details, and information for consumers. The recommendation model improves the efficiency and effectiveness of E-commerce applications that provide better performance and services for users (Coussement et al., 2021). A classification method-based recommendation model is also used in E-commerce, which identifies customers’ requirements by analyzing the previous activities of users. The knowledge discovery technique is used in the classification method to determine the personalities and characteristics of products and provide an optimal set of data for customers in E-commerce applications (Chen et al., 2021b).

Emotion analysis is a process that identifies and analyzes the emotions expressed in text format. The emotion analysis process extracts the exact meaning of data and understands the emotion given by the users (Chung and Zeng, 2020). The emotion analysis process plays a major role in various fields that determine the meaning of a text by performing an analysis process. Emotion analysis is most commonly used in the E-commerce recommendation model that provides an important set of data that provide better services for users (Grigorios et al., 2022). The emotion analysis process improves the quality and performance of services that E-commerce recommendation models provide. A multi-classification method is used in the emotion analysis process, identifying various emotions and providing exact details about each text and message (Meng et al., 2021). Emotions are classified based on previously recorded details and produce a final set of data for the communication and interaction process in E-commerce. The multi-classification method identifies emotions such as happiness, anger, disappointment, and misunderstanding. Multi-classification increases the accuracy rate and efficiency rate in the emotion analysis process, which indicates the actual meaning of text formats (Zhao et al., 2019; Kang et al., 2021).

Machine learning (ML) techniques are mostly used in the detection, prediction, and analysis processes, improving the overall accuracy rate in the prediction process. The ML technique is also used in the E-commerce emotion analysis process to identify the customers’ actual meaning of text and messages (Demircan et al., 2021). The convolutional neural network (CNN) algorithm is used in the emotion analysis process, which predicts and detects the exact emotions of users. The feature extraction process is used in CNN, which extracts customers’ exact emotions and feelings in an E-commerce application (Xu et al., 2020). CNN combines the current emotions with previously recorded emotions and finds out the exact meaning of the user’s emotions. A gated recurrent unit (GRU) is also used in the E-commerce emotion analysis process that extracts the important emotions and details from textual messages (Ghavamipoor and Hashemi Golpayegani, 2020). The attention mechanism is used in GRU to classify the weight of emotions and produce the text’s actual meaning, which enhances the efficiency rate of E-commerce applications. A support vector machine (SVM) algorithm is commonly used in the E-commerce emotion analysis process that predicts the emotion based on a certain set of features. SVM collects the necessary data from the database and provides exact sentiments and emotions of customers in E-commerce applications (Guo, 2020; Xu et al., 2022).

## Related Works

Wang H. et al. (2020) introduced a domain recommender method using a deep neural network (DNN) approach for E-commerce. The main goal of the proposed method is to solve the cold-start problem presented in the online shopping application. DNN compared the domains and users to find out the problems and provide optimal solutions to cold-start problems. The proposed domain recommender method reduces the problems and provides various services for users in online shopping. The proposed method increases the effectiveness and efficiency of E-commerce.

Chen et al. (2021a) proposed a new storytelling recommendation method for E-commerce platforms using an additional domain transfer network. The proposed method is mainly used to identify the interests and searches of users and then produce an accurate set of data for a service provider. First, user behavior patterns are identified using an additional domain transfer network, and domain gaps are identified by the domain transfer network and produce effective details about each domain. The proposed method reduces the error rate in service providers and improves the feasibility of E-commerce.

Gao et al. (2020) introduced a triad-based word-of-mouth recommendation (TriM) model for socialized E-commerce applications. In E-commerce, the user sends product links to other persons and promotes the products. The proposed TriM model finds out both user and receiver details and produces an optimal dataset for the analysis process. The produced data are used to identify the interests of users in E-commerce. The TriM model improves the performance and efficiency of providing services for users in social E-commerce applications.

Ji et al. (2019) proposed a hybrid recommendation model for social E-commerce. Ratings, reviews, requests, interests, and searches are collected for the data analysis process. The recommendation model detects the actual interest of users based on previously recorded data and provides necessary services to the users. A regression algorithm is used here to identify users’ features and behavior patterns for the recommendation process. Experimental results show that the proposed method improves the effectiveness and performance of the E-commerce system.

Wang and Qiu (2021) proposed a new DNN model using side information for the fashion collaboration recommendation process in E-commerce. Purchase data, product details, textural description, and user interest are collected and analyzed using the DNN model. The fashion collaboration recommendation model identifies users’ experience with products and services provided by E-commerce. The proposed model improves the efficiency and feasibility of E-commerce by reducing the latency rate in product searching.

Islek and Oguducu (2022) introduced a hierarchical recommendation model using online user reviews for E-commerce platforms. Textural information of the products is identified by bidirectional encoders and produces an optimal set of data for a service provider. The hierarchical recommendation model provides a transparent system for the users, which improves the trustworthiness of the platforms. The proposed method increases the accuracy rate in the identification process, which improves the performance of the E-commerce system.

Wijaya (2022) proposed a conceptual model for E-commerce using a recommendation system. Product details and reviews are filtered, producing an actual dataset for the recommendation process. Hyper-personalization is used here to provide optimal recommendations for users in E-commerce. The proposed model increases conceptual products’ overall efficiency and effectiveness in online shopping platforms.

Wang K. et al. (2020) introduced a personalized recommendation analysis process for E-commerce using a deep-learned clustering approach. A deep learning clustering algorithm is used here to analyze the data needed for the recommendation process in online shopping. The clustering method identifies users’ patterns, interests, and behavior. Product information, location, and delivery time are recommended for users by the proposed analysis process. The proposed recommendation analysis method improves the effectiveness of E-commerce.

Zheng et al. (2021) proposed a recommendation model for E-commerce using heterogenous type-specific information. The user’s latent features and patterns are identified for the recommendation process. The proposed recommendation model provides necessary services and products for users with an effective set of details. Experimental results show that the proposed model increases the accuracy rate in the recommendation process and improves the system’s efficiency.

Mao et al. (2019) introduced a hypergraph ranking method for the recommendations process in E-commerce. The ranking method identifies users’ searches, interests, requests, and feedback. The proposed ranking method provides a feasible dataset for the recommendation process. Multi-objective recommendations and searches are collected and stored for further analysis process. The proposed model provides effective details and products for users through a recommendation process in the E-commerce system.

Xia et al. (2021) proposed an outlier data mining-based recommendation model for E-commerce. The proposed model is mainly used for the knowledge management process and provides an optimal dataset for the analysis process. A filtering algorithm is used to fetch the necessary data for the recommendation process. The data mining approach finds out the details of the recommendation process and provides data for the service provider. The proposed model improves the efficiency and effectiveness of E-commerce.

Li et al. (2022) introduced a new recommendation model using a hybrid recommendation algorithm for E-commerce. In an online shopping platform, image content information is collected, containing details about users’ reviews and requests. A classification method is used here to classify the details and produce the final dataset for the recommendation process. Image content and features are necessary for providing an effective dataset for the recommendation process. The proposed recommendation model reduces the latency rate in the search process and improves the accuracy rate in recommending goods to the users.

Zhou (2020) proposed a deep learning-based recommendation model for E-commerce. Deep learning is used here to predict users’ interests and searches, providing an effective recommendation process details. Users’ expressions over products and goods are also identified using certain filters. The identified data plays a vital role in the recommendation process that finds out the actual interest of users in online shopping platforms. The proposed model improves the accuracy rate in the recommendation process that reduces the complexity and latency rate in the analysis process.

Ding et al. (2019) introduced a user-centered recommendation-based dynamic graph model for E-commerce. A user clustering algorithm is used to identify the necessary dataset for the recommendation process. The clustering algorithm identifies preference and interest patterns and produces a feasible dataset for the dynamic graph model. Compared with other models, the propped graph model increases the accuracy rate in the recommendation process.

## The Proposed Syntactic Data Inquiring Scheme

**FIGURE 1:**
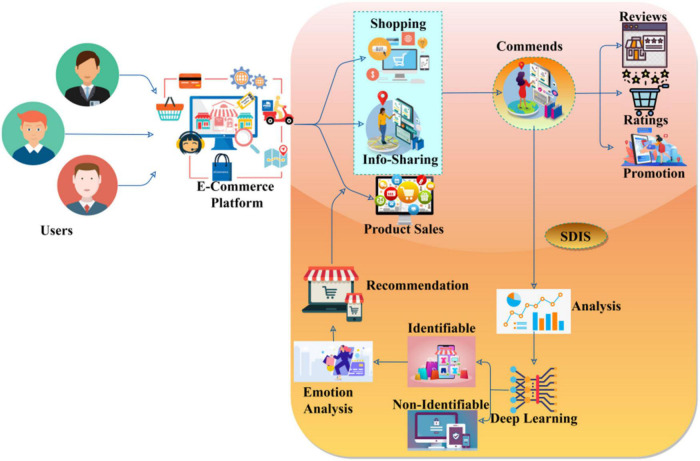
SDIS.

A Syntactic Data Inquiring Scheme (SDIS) is designed to improve the user relationship with the E-commerce platforms. The user-platform relationship analysis is based on diverse user feature inputs of digital platforms. The user experience and connection between E-commerce platforms are required from the semantic emotions and the personalized recommendation observed in different time instances through user shopping and information sharing based on the product sales in the E-commerce platform. The semantic emotional analysis is based on digital user comments and repetition on the E-commerce platforms. Figure 1 portrays the proposed SDIS.

The main role of this scheme is to reduce negative comments and repeated comments on decentralized E-commerce platforms. The risk factor in this work is a classification of identifiable and non-identifiable emotional data analysis recommendations for the new media era with the new user comments and user relationship instances. The users’ comments are stored in the form of reviews, ratings, and promotions from the previous user emotion data based on positive and repeated comments. The multi-resource access and information sharing require a recommendation system based on conventional user services for buying products on E-commerce platforms (refer to Figure 1). The shopping activities on digital platforms are accessed through user relationships, and they provide best-affordable user services through promotions, ease of access, product selection, etc. The consecutive user emotion data are classified as identifiable and non-identifiable. Service recommendations and further promotions reduce the prioritization and product sales in shopping on digital platforms. The role of this modeling is to increase the prioritization of user requirements on the E-commerce platform and encounter new comments based on user interest and recommendation factors through a deep learning process. The consecutive shopping and information sharing and product sales result in disadvantages such as security issues, a lack of personal touch, no guarantee about product quality, a long delivery period, and not trying before buying and advantages such as a faster buying process, elimination of operating cost, detailed product information, personalized shopping experience, connecting far and wide, available 24 × 7, and retargeting the customer for shopping products on E-commerce platforms and based on the semantic emotion data analysis and user recommendation, respectively, then


(1a) $$\max\,\sum_{t=\alpha }U_{E\,c}\,{\left(S_{h},I_{s}\right)}^{\alpha }$$

Such that


(1b) $$\sum_{i\in U_{E\,c}}S_{h}=\prod_{t=\alpha }S_{h}-1-\left[\frac{P_{s}}{\sum \left(S_{H}+I_{S}\right)}\right]$$

where the variables *U*_*Ec*_, *S_H_*, *I_S_*, and *P*_*s*_ are used to represent the E-commerce platform users, shopping, information sharing, and product sales based on user experience and connection through semantic emotions. The condition *U*_*Ec*_(*S*_*h*_,*I*_*s*_) is used to represent the semantic emotion data availability based on digital platform *i* connection between shopping and information sharing at different time intervals *t*. The maximum process α = 1 achieves high sequential service sessions *R*_*c*_ for the service recommendation to the cloud resources. Instead, the variables *S*_*h*_, *I*_*S*_, and *P*_*s*_ are not ideal due to *t* as α ∈ [0,1] is the differing condition. Instead, α = 1 is not ensuring the shopping and information sharing time intervals $S_{H}^{t}$ and $I_{S}^{t}$, resulting in a delay in product sales. This problem is referred to as hardness in shopping on digital platforms in personalized recommendation scenarios. In this E-commerce platform, user experience and the connection depend on the user comments analysis, and deep learning is used jointly in this proposed scheme for maximizing product distribution ratio through E-commerce platforms.

## Semantic Emotion Data Analysis

In an E-commerce platform based on semantic emotion data analysis, the personalized recommendation and user categories across the digital platforms are observed by shopping and information sharing from the diverse user features. Initially, this proposed scheme identifies the new emotional data based on user comments and repetition in the SDIS-assisted E-commerce shopping. The user comments such as reviews, ratings, and promotions are required based on product features, requirements, and quality. Hence, the emotional data analysis, based on online user comments and repeated comments, relies on the user relationship and connections as in Equation (1). The probability of shopping and information sharing depends on product sales in *U*_*Ec*_ without negative comments, and therefore, ρ(*EC*_*ED*_) is discussed in the following Equation:


(2) $$\rho \,\left(E\,C_{E\,D}\right)=\frac{\prod_{t=U_{E\,c}}S_{h_{\alpha }}}{\prod_{t=\alpha }U_{E\,c_{\alpha }}+\sum \left(S_{h},I_{s}\right)}\,i^{-\frac{S_{H}^{t}*I_{S}^{t}}{C^{m}}.R.\Delta .\alpha }$$

where the variables *C^m^* and *R* represent the user comments and relationships, respectively, based on the emotional data in the shopping and information sharing process at different time instances. $S_{H}^{t}$ and $I_{S}^{t}$ are the availability of the diverse E-commerce user features based on user relationship and connection with the online shopping platforms, respectively. In particular, the user comments and repetition based on shopping products expression of $1-\left[\frac{P_{s}}{\sum \left(S_{H}+I_{S}\right)}\right]$ is estimated using Δ. $S_{H}^{t}$, $I_{S}^{t}$, and *C^m^* are used to compute a solution for both digital shopping and information sharing based on *P_s_*. This product’s availability is based on user comments, and repetition identifies the emotional data. This semantic emotion analysis is classified as identifiable and non-identifiable data based on positive comments, and repeated comments through the deep learning paradigm are estimated as follows:


(3) $$P_{s}\,\forall \alpha \in S_{H}^{t}\,\cup I_{S}^{t}=\left[\left[\left(1-\Delta \right)\,\frac{P_{s}}{I_{S}}\right]-\left[t*\frac{P_{s}}{i}-\left(I_{S}-I_{S}^{\Delta }\right)\right]\right],i\in C^{m}$$

**FIGURE 2:**
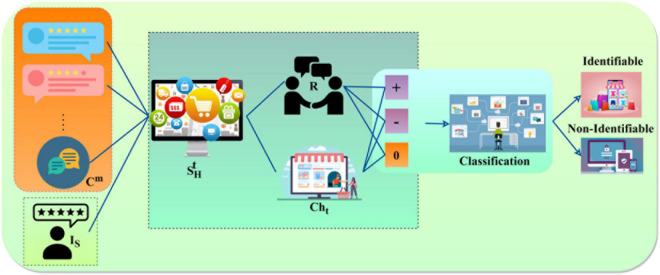
Comment (information) classification progression.

Based on the above Equation, the emotional data availability based on the user comments and repetition *C^m^* are not modified by other users during $S_{H}^{t}$ or $I_{S}^{t}$ with the E-commerce platform. The classification of user comments in the initial and final stages is accessed for maximizing Δ. In this condition, $P_{s}\,\forall \alpha \in S_{H}^{t}\,\cup I_{S}^{t}$ exceeds, and then an emotional data analysis is performed based on service recommendations with digital shopping. E-commerce-based products shopping holds the user relationship and comments *U*_*Ec*_ and security service of *C^m^* as (α, *U*, *U*^Δ^, ρ(*EC*_*ED*_)) recommend the new products *I*_*S*_ or modify the user requirements *U*_*Ec*_ in all the $S_{H}^{t}$ and $I_{S}^{t}$. Figure 2 displays the comment (information) classification progression. The classification progression relies on the frequency $\left(S_{H}^{t}\right)$ and *C^m^* provided in *t*. This is mapped for *R* and *Ch*_*t*_; if *Ch*_*t*_ varies, and then *Ch*_Δ_ also varies and changes the comment type. The *R* between the E-commerce platform and the users is modified based on negative or positive, or unidentifiable comments (post-classification) based on *P_S_*∀α and *U_C_ m*. In this learning process, *Ch*_*t*_ and ρ(*EC*_*ED*_) are required for *C^m^* and *R* classification (refer to Figure 2). This output is considered for product recommendations for new products and requirement changes based on user needs. In this manner, the outputs are predictive using prioritization and training, and it depends on *U*_*Ec*_ and α for $\sum_{t\in U_{E\,c}}U_{E\,c}=U$, *U*^Δ^ and ρ(*EC*_*ED*_) for the instance of condition in the above-discussed equation. Let *Ch*_*t*_ and *Ch*_Δ_ denote the modifications in user requirements and relationship *U*_*Ec*_ in the initial stage. It refers to the new emotional data analysis and product requirement changes for the user relationship with E-commerce platform that relies on different user features and experience. Therefore, the overall user comment’s analysis *U*_*C^m^*_ is given as follows:


(4) $$U_{C^{m}}=I_{S}+C\,h_{t}+C\,h_{\Delta }$$

Such that


(5a) $$I_{S}=C\,h_{t}\,\left(S_{H}^{t}*I_{S}^{t}-\frac{U_{R}}{U_{E\,c}}\right)+C\,h_{\Delta }\,\left(S_{H}^{t}\right)\,\forall i\in S_{H}^{t}\,a\,n\,d\,i\in C^{m}$$

where


(5b) $$\begin{array}{c} C\,h_{t}=\sum_{i\in U_{E\,c}}U_{E\,c}=n\times \sum_{i\in U_{E\,c}}\frac{U_{E\,c}}{U_{R}}=n\times \sum_{t\in U_{E\,c}}S_{H}^{t}\\ a\,n\,d\\ C\,h_{\Delta }=\sum_{i\in U_{E\,c}}U_{E\,c}^{i}-\left(1-\Delta^{i\,j}\right)=\sum_{i\in I_{S}}\left(U_{R}-I_{S}^{\Delta }\right) \end{array}\}$$

From Equation (4), *U*_*C^m^*_ is computed as a circumstance of $U_{E\,c}^{i}$ and *U* withΔ to find the exact output. Therefore, these semantic emotion data are responsible for user positive comments and repeated comments based on a deep learning paradigm depending on the recommendation system, strengthening the user relationship with fewer negative comments and delays in product delivery. The user relationship is designed for the training instance of user interest and recommendation factor to prioritize user requirements. The priority based on user requirements can be available for the same or another user with the E-commerce platform for next time. Based on this consecutive process, the conventional recommendation factor provides comments to sequential user interest consisting of *U*_*Ec*_ and *I_S_*. The user interests are used in both emotional data analysis and recommendation factors by verifying priority and user interest with the help of an E-commerce platform.

## Recommendation Factor

The current user interest and classified recommendation based on shopping with the E-commerce platform probabilistic recommendation factors *C^m^* and ρ(*I*_*S*_) analyzed for further promotions and positive comments on the digital platform. In particular, the diverse user features and requirements based on the above conditions are analyzed to prioritize user requirements to maximize product availability. The probability of prioritization of user requirements *p*_*r*_ is estimated as follows:


(6) $$\rho \,\left(p_{r}\right)=\frac{\rho \,\left(C^{m}\,\cap I_{S}\right)}{\rho \,\left(I_{S}\right)}$$

**FIGURE 3:**
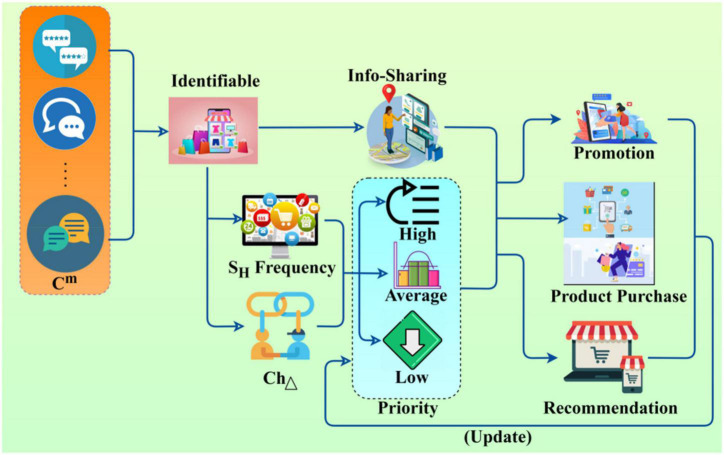
Prioritization process flow.

The recommendation factor is based on identifiable and non-identifiable emotion data using user comments and repetition analysis processed through deep learning. This learning identifies user interests and requirements in both shopping and information sharing instances. The above condition is analyzed with the positive comments and repeated comment sequences using a recommendation system. The user relationship depends on requirements for identifying the reviews, ratings, and promotions probabilities during the training process. Hence, the conditions for user recommendation factors are classified and trained, which follows user experience through the prioritization of user requirements. The prioritization process flow is presented in Figure 3. In Figure 3, the priority determining process flow is presented based on *S_H_* and *Ch*_Δ_. The output is classified as high, average, and low based on *Ch*_Δ_∀*R* and hence the *U*_*C*_*m* ∈ *I*_*S*_ is used for promotion, purchase, and recommendations. These features are filtered for updating the priority. The prioritization of user requirements is prescribed for both the user experience and connection by computing the probability of prioritization of user requirements in different instances. The user requirements *p*_*r*_(*req*) depend on maximum promotions(*t*_*i*_). In this prioritization of user requirements and further promotion analysis, computing is performed for positive comments and repeated comments balancing. To reduce the negative comments, the modified user relationship of *R_L_* is given as follows:


(7) $$U_{E\,c}\,\left(R_{L}\right)=U_{E\,c}\,\left[\frac{\left(p_{r}\times \frac{S_{H}}{I_{S}}\right)}{r\,e\,q\,\left(U_{E\,c}\right)-S_{H}^{t}+I_{S}^{t}\,\left(t_{i}\right)}\right]$$

In Equation (9), the E-commerce platform users’ shopping and information sharing process instances are analyzed, and prioritization of user requirements based on recommendation factors and requirements as per the users’ interests is either of *U*_*Ec*_or *p_r_*, in both instances, if *req*(*U*_*Ec*_) = 0, then the semantic emotion data is identified as *U*_*Ec*_ = *I*_*S*_ = *p*_*r*_ is the maximizing recommendation condition, and if *req*(*U*_*Ec*_) = 0 = 1, then *I*_*S*_ = *p*_*r*_−*U*_*Ec*_ and *I_S_* = *p*_*r*_. Hence, the occurrence of *p*_*r*_ = *I*_*S*_ is a reliable solution, whereas the minimization of negative comments for all the shopping and information sharing with the E-commerce platform is as derived in Equation (1). This condition is analyzed for all *U*_*Ec*_ ∈ *t*_*i*_ and α ∈ *R*_*L*_ in Equation (1). The recommendation factor in this scenario is trained for further promotions, where user experience and connection are exploited through semantic emotion data, and therefore, the comments are monitored as per Equation (1). In any instance of *p_r_*, if the condition is analyzed *I_S_* < *p*_*r*_, then the user gives comments for the products, which again outputs in the recommendation factor. Based on further promotions, the user interest (*R*_*L*_) in both the conditions, i.e., ρ(*I*_*S*_) and ρ(*p*_*r*_), is computed in the prioritization manner of *p*_*r*_ in order to ensure *I*_*S*_ > *p*_*r*_ as given in the following equation:


(8) RL[ρ(ISpr)]=12πΔexpression[(IsSH−ISΔ)×(IS−Δ)pr22Δ],∀pr∈α in ti

In the above Equation, the consecutive manner of user-platform relationship and requirements consideration of *U*_*EC*_ as a diverse user feature of *t*_*i*_ and $\left[t_{i}-\frac{S_{H}}{I_{S}}\right]$ instances, the above user interest consideration based on prioritization is estimated for *t_i_*. Instead, the promotions of either the shopping and information sharing for α in any *t_i_* are identifying recommendation factors for maximizing *U*_*EC*_. From the condition, the promotions/recommendations based on ρ(*I*_*S*_) and ρ(*p*_*r*_) factors.

The computation of semantic emotional data analysis for all the E-commerce platforms, based on the recommendation factors, differentiates the identifiable and non-identifiable emotional data through product sales for *U*_*EC*_ from *I*_*S*_ ensuring. The user experience and diverse user features are available during shopping and information sharing. Therefore, the user requirements are based on Δ other than the next user *U*. From this condition, if the positive comments and repeated comments increase the promotions and recommendations, the minimum negative comments and delivery delays are attuned. Therefore, the user comments and repetition consecutively maximize E-commerce platforms through shopping products online; it finds the negative comments in those digital platforms and resolves the issues with reply comments through deep learning. The identifiable emotion data is the process for further promotions in the E-commerce platforms based on user interest. This recommendation system under E-commerce platform shopping is used to reduce negative comments and non-identifiable emotions.

## Discussion

**FIGURE 4:**
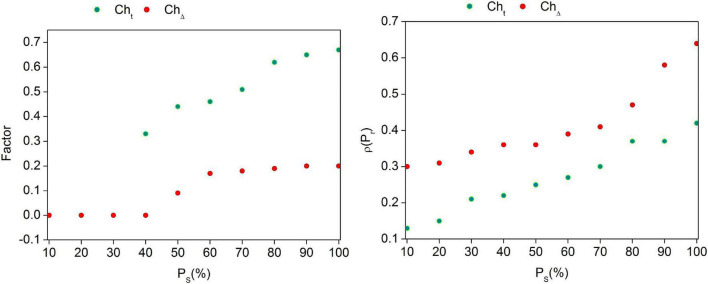
*PCh_t_*, *Ch*_Δ_, and ρ(*p*_*r*_) for the varying *P_S_* (%).

The proposed scheme’s analysis is discussed in this section using datasets and comparative validations. The Amazon dataset form is used for validating the recommendation, priority, and emotional relationship between the buyers/users and the E-commerce platform. This dataset provides eight-column information related to purchase categories, reviews, products, sentiment, and date. The training is performed using 4,001 records, of which 1,000 data entries are tested using the proposed scheme. In Figure 4, the analysis for *Ch*_*t*_, *Ch*_Δ_, and ρ(*p*_*r*_) for the varying *P_S_* (%) is presented.Figure 4 presents the analysis of *Ch*_*t*_, *Ch*_Δ_, and ρ(*p*_*r*_) for the varying *P_S_*(%). As the data analysis rate increases, the *Ch*_*t*_ becomes independent of *Ch*_Δ_ in *I_S_*. This is required for *R* and *R_L_* variation analysis due toα. In this context, the non-identifiable data (comment/recommendation) are prevented from impacting the new *U*_*EC*_(*R*_*L*_). Therefore, either *Ch*_Δ_ or *Ch*_*t*_ is alone impacted and hence the variation. Contrarily, as *Ch*_Δ_ rises, the ρ(*p*_*r*_) is analyzed for preventing *P_S_* lag. Deep learning is independently trained under varying *C^m^* and *P*_*S*_∀α for preventing negative *R_L_*impacts. In that case, the promotional recommendations are performed. This reduces the modification rate impacts over the varying *I_S_* ∈ *U*_*C*_*m*. The analysis for *P_S_* (%) for the varying *S_H_* and products is presented in Table 1.

**TABLE 1:** *P*_*s*_ (%) for the varying *S*_*H*_.

*S*_*H*_	Products	Comment		Recommendation		*P_S_* (%)
				
		Yes	No	Yes	No	
2	1	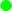		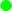		51.03
4	3		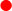		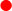	85.59
6	4	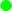		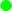		58.69
8	6		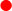		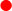	80.65
10	10		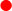	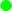		62.25
12	8	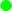		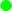		68.454
14	9	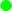		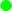		61.25
16	4	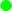			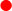	79.41
18	2		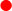		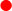	75.36
20	8	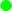		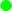		68.48

In Table 1, the *P_S_*(%) for varying *S_H_* for different products are tabulated. The green marking represents the user’s commented/recommended “Yes.” This is estimated for the comments and recommendations given products from the dataset. The impact of *Ch*_Δ_ increases the chance for *P_S_*(%) for preventing *P_S_* lag, and therefore, the analysis shoots up if the recommendation is negative. Contrarily, if *Ch*_*t*_ is suppressed using *U*_*EC*_, then *R* = *R*_*L*_ is verified. Therefore, *P_S_*(%) is augmented with the modification factor for preventing lags. The prioritization is performed based on the training dataset’s keywords that classify the emotions as positive/negative. Based on the ρ*_S_* ∀ α, the semantic data vary such that *U_C_m* recommends (or discards) *Ch*_*t*_ and *Ch*_Δ_. In Table 2, the prioritization process from the input dataset is portrayed.

**TABLE 2:** Prioritization process from the input dataset.

Products	Data keywords	Comments	*S*_*H*_	Actual	Testing
Audio systems	Great, recommended, Excellent, loved it.	85	15	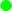	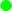
Home theaters	Upgraded, wow, awesome, best, outstanding, perfect	103	20	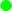	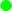
Computers and accessories	Useless, the worst thing failed, weak	18	2	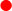	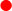
Tablets	OK works decent	24	5	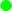	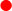
TV	Cool, perfect, works, best, easy	55	9	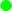	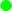
Networking and electronics	Fragile, useless, worst thing, weak, but works	80	16	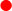	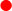
E-Books and E-Readers	Loved it book lovers, recommend, awesome, outstanding, excellent, love it	99	19	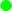	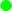
Home and tools	Not worth to money, works decent, OK weak	45	12	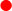	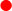
Back to college	Perfect, recommend, awesome, OK	39	8	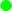	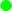
Walmart deals	Great, wow, decent, cool, perfect	75	17	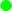	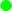

**FIGURE 5:**
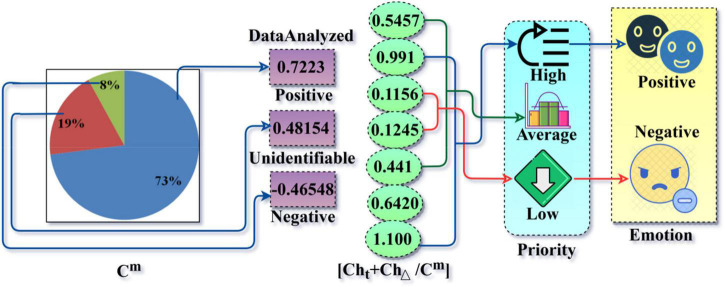
*R* state (emotion) identification from the SDIS output.

Based on SDIS, the priority for the input is classified as high, average, and low. This is classified based on $P\,o\,s\,i\,t\,i\,v\,e\,\frac{r\,e\,v\,i\,e\,w\,s}{\left(p\,o\,s\,i\,t\,i\,v\,e+n\,e\,g\,a\,t\,i\,v\,e\right)\,R\,e\,v\,i\,e\,w\,s}$ of the products listed in Table 2. The emotion is classified as positive/negative, and its priority is estimated using Equation (6). Based on these factors, the *R* state is identified from SDIS output, as presented in Figure 5.The *C^m^* observed is split into positive, negative, and unidentifiable with its corresponding ratio (refer to Figure 4). This is observed based on the data analyzed from user comments. In the comment rate for priority classification, high (0.6–1), low (0–0.4), and average (0.4–0.6) are mapped from which the emotion is detected (refer to Figure 5). Based on this dataset information, the metrics recommendation, data analysis ratio, and modification rates are comparatively analyzed alongside RHM (Ji et al., 2019), MRHR (Mao et al., 2019), and PR-DLC (Wang K. et al., 2020) methods discussed in Tables 3, 4. The *S_H_* and *C^m^* vary in this comparative analysis.

**TABLE 3:** Comparative analysis for varying *S*_*H*_.

Metrics	RHM	MRHR	PR-DLC	SDIS
Recommendation	0.372	0.452	0.627	0.783
Data analysis ratio	58.72	65.2	73.57	84.816
Modification rate	0.44	0.39	0.32	0.25

Finding: The proposed SDIS maximizes recommendation by 14.97%, the data analysis ratio by 9.49%, and reduces the modification rate by 13.3%.

**TABLE 4:** Comparative analysis for varying *C*_*m*_.

Metrics	RHM	MRHR	PR-DLC	SDIS
Recommendation	0.323	0.429	0.502	0.569
Data analysis ratio	59.11	65.02	75.14	85.244
Modification rate	0.43	0.38	0.33	0.21

Finding: The proposed SDIS maximizes recommendation by 15.1%, the data analysis ratio by 9.41%, and reduces the modification rate by 17%.

### Recommendation ratio

**FIGURE 6:**
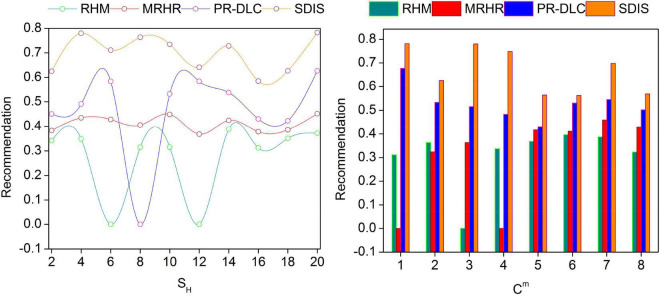
Recommendation comparisons.

Figure 6 presents the analysis of recommendation factors for varying *S_H_* and *C*_*m*_. The chances for *S_H_* and *C_m_* based ρ(*EC*_*ED*_) is unpredictable due to *R* estimation. This is balanced by using the deep learning to reduce the *C*_*m*_ density; the unidentifiable *C_m_* is reduced from the *P*_*S*_∀α. In the semantic data analysis process, *U*_*EC*_(*S*_*H*_, *I*_*S*_) is estimated usingα ∈ [0,1] range for preventing negative commands. If the negative commends increase, then *P*_*r*_-based recommendations are delivered to prevent non-recommendation or offensive feedback from the users. From the *U*_*Cm*_, *I*_*S*_ required for *Ch*_Δ_ and *Ch*_*t*_ validation is performed for different *S_H_*. Contrarily, *U*_*EC*_(*R*_*L*_) is analyzed for improving the recommendation for the successive *S_H_*. Therefore, the ρ(*p*_*r*_) is identified for preventing fluctuating non-recommending sessions. The deep learning process then (post unidentifiable *I_S_* mitigation) is trained using *R_L_* as in Equation (8). Therefore, the negative feedback is suppressed from distinct*Ch*_Δ_ + *Ch*_*t*_; the further recommendation is preceded using ρ(*EC*_*ED*_). Hence, the recommendations are exploited from the previous training instances for preventing *P_S_* downfall.

### Data analysis ratio

**FIGURE 7:**
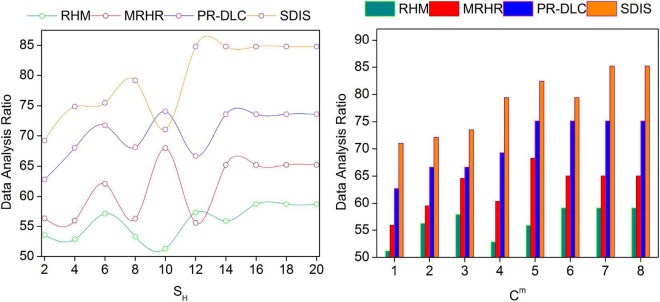
Data analysis ratio comparisons.

The proposed scheme achieves high data analysis for the varying *S_H_* and *C*_*m*_ as illustrated in Figure 7. The data analysis is performed separately for the considered *x*-axis variants. First, the conventional *C_m_*-based *P*_*S*_∀α is analyzed to prevent lag in analysis. In the second semantic data analysis, the *Ch*_*t*_- and *Ch*_Δ_-based data is handled. In the learning output, priority and *I*_*S*_-based assessments are performed for augmenting new recommendations. The contrary process is based on *U*_*EC*_(*S*_*H*_, *I*_*S*_) is required for preventing unidentifiable data analysis. The consecutive learning process estimates *I_S_* as in Equation (5a) for reducing the analysis. The consecutive learning process estimates *I*_*S*_ as in Equation (5a) for reducing the analysis mitigation. Further analysis∀(*R*.Δ) is performed before the classification, so the learning requires identifiable data. For the varying *U* and *U*^Δ^ identified from the training sessions, the data analysis is improved based on *R*_*L*_ and its modifications for preventing *Ch*_Δ_. Therefore, the data under varying *C_m_* and *R* increase the data analysis ratio.

### Modification rate

**FIGURE 8:**
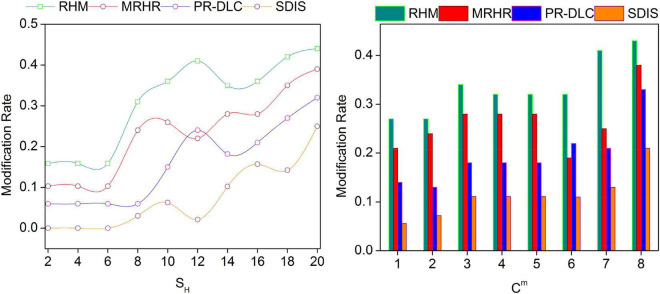
Modification rate comparisons.

The proposed scheme achieves less modification rate by classifying *Ch*_Δ_ and *Ch*_*t*_ for different *C_m_* and *S_H_*. In the classification, the non-identifiable data are discarded to prevent implied modifications on *R*. This sustains the initial negative feedback/comments by discarding the same. Contrarily, the impact of *I_S_* over the *p*_*r*_ is reduced by analyzing *U*_*EC*_(*R*_*L*_) such that the modifications are confined. Based on the varying sessions and user comments, the data augmentation increases, for which modification is reduced. This is instigated under different *P_S_*∀α such that *C_m_*∉ρ(*EC*_*ED*_) and hence *P_r_* is the consecutive process. The *S*_*H*_ based on training data variates, the modification instances due to which the *Ch*_Δ_ impact is confined∀*Ch*_*t*_. The recurrent deep learning process consecutively validates *U_C_m* ∈ *I*_*S*_ such that ρ(*p*_*r*_) is high for *Ch*_*t*_-induced data. In further identification∀*R* = *R*_*L*_, the modification is less, and hence appropriate data analysis is performed. Therefore, the proposed scheme reduces the modification rate of *R* for the varying *S_H_* and *C*_*m*_ (refer to Figure 8). The above comparative analysis results are tabulated in Tables 3, 4 for the varying *S_H_* and *C_m_*.

## Conclusion

This study presents a SDIS for semantic emotional data analysis in E-commerce platforms. This scheme aims to improve the user’s shopping experience through better recommendations and promotions. The user-shared data such as reviews, comments, ratings, and recommendations serve as the input for this scheme in analyzing the relationship with the E-commerce platform. The input data is classified using a deep learning paradigm to prevent the unidentifiable data impact from degrading the recommendations. The post-classification process and the prioritization-based relationship modifications are suppressed thereby improving the recommendations. The priority probability is analyzed based on the unavoidable modifications by training the network through previous classifications. In this iterated process, the maximum outcome is achieved if the classified modification factor does not impact the relationship in either case. Therefore, the learning paradigm is trained using modified relationship data to provide the best affordable E-commerce experience. From the experimental analysis, as the session frequency increases, the proposed SDIS maximizes recommendation by 15.1%, increases the data analysis ratio by 9.41%, and reduces the modification rate by 17%.

## Data availability statement

The original contributions presented in this study are included in the article/supplementary material, further inquiries can be directed to the corresponding author/s.

## Author contributions

YL: writing. ZD: transferring. Both authors contributed to the article and approved the submitted version.

## Conflict of interest

The authors declare that the research was conducted in the absence of any commercial or financial relationships that could be construed as a potential conflict of interest.

## Publisher’s note

All claims expressed in this article are solely those of the authors and do not necessarily represent those of their affiliated organizations, or those of the publisher, the editors and the reviewers. Any product that may be evaluated in this article, or claim that may be made by its manufacturer, is not guaranteed or endorsed by the publisher.
